# Preirradiation of Spheroids with ^225^Ac-Trastuzumab Improves Penetration of ^225^Ac-Liposomes and MIRDcell Predictions of Responses to Drug Cocktails

**DOI:** 10.2967/jnumed.124.269273

**Published:** 2025-08

**Authors:** Hima Tallam, Rajiv Nair, Aira Sarkar, Sumudu Katugampola, Stavroula Sofou, Roger W. Howell

**Affiliations:** 1 Department of Radiology and Center for Cell Signaling, New Jersey Medical School, Rutgers University, Newark, New Jersey;; 2 Department of Chemical and Biomolecular Engineering, Institute for NanoBioTechnology, Johns Hopkins University, Baltimore, Maryland; and; 3 Sidney Kimmel Comprehensive Cancer Center, Cancer Invasion and Metastasis Program, Department of Oncology, Johns Hopkins University, Baltimore, Maryland

**Keywords:** radiopharmaceutical therapy, micrometastasis, ^225^Ac-liposome, ^225^Ac-trastuzumab, dosimetry

## Abstract

This investigation examined the factors involved in predicting the responses of micrometastases to targeted ^225^Ac-based therapies and in optimizing these therapies through the use of cocktails of radiopharmaceuticals (RPTs). **Methods:** MIRDcell version 4 was used to model the surviving fraction (SF) of cells in multicellular spheroids that were treated with cocktails of ^225^Ac RPTs as previously reported by Howe et al. in this journal. Spheroids were treated with varying activity concentrations of [^225^Ac]Ac-DOTA-SCN-trastuzumab (^225^Ac-trastuzumab) and ^225^Ac-DOTA–encapsulating liposomes (^225^Ac-liposomes). With the total activity concentration kept constant at 13.75 kBq/mL, 5 different activity distributions among the liposomes and antibodies were evaluated: 0%, 30%, 50%, 70%, and 100% of the total activity on each carrier. Penetration of the ^225^Ac-trastuzumab and ^225^Ac-liposomes into the spheroids was obtained with fluorescent surrogates in the previous work and remeasured here to determine whether preirradiating the spheroids with ^225^Ac-trastuzumab affected the spatiotemporal distribution of the liposomes. These data were used to compare MIRDcell-predicted SFs with experimental spheroid outgrowths. In addition, the artificial intelligence tool in MIRDcell was used to optimize the best cocktail formulation of ^225^Ac-antibodies and ^225^Ac-liposomes to achieve SFs of less than 0.0001 while minimizing the number of total decays necessary. **Results:** The penetration profiles of spheroids with 200-µm radii show a 42% increase in the total number of decays attributed to ^225^Ac-liposomes between 0 and 100 µm from the centers of spheroids when first pretreated with 6.5 kBq/mL ^225^Ac-trastuzumab. The MIRDcell predictions based on liposome penetration data obtained after preirradiation with ^225^Ac-trastuzumab also provided a better match to the experimental data. Artificial intelligence optimization found that although ^225^Ac-liposomes alone are able to sterilize all the cancer cells in the spheroid, 44% more total decays are required than when using a cocktail of ^225^Ac-trastuzumab and ^225^Ac-liposomes. **Conclusion:** Penetration of ^225^Ac-liposomes was enhanced by pretreatment with ^225^Ac-trastuzumab, and strategies to optimize penetration into micrometastases are important for RPT therapy with carrier cocktails. RPT cocktails, as opposed to single agents, may be required to eliminate circulating tumor cells, disseminated tumor cells, and micrometastases.

## Visual Abstract

**Figure:**
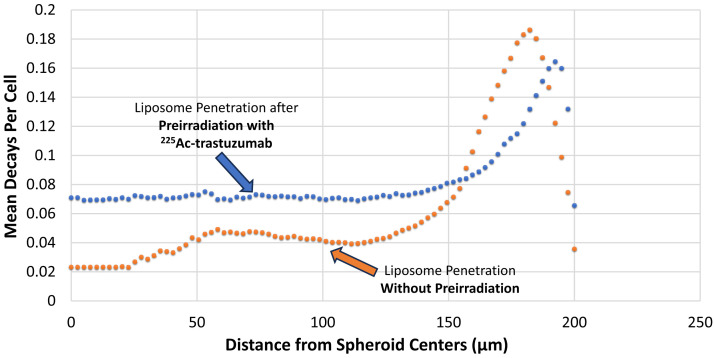

As development of radiopharmaceuticals (RPTs) gains popularity, there is increasing interest in using α-particle–emitting RPTs in the treatment of microscale disease (1*–*3). Single traversals of α-particles through the cell nucleus cause numerous double-strand DNA breaks, bypassing constraints associated with radiation and chemical resistance in micrometastases (4). They are particularly well suited for microscale disease as they can travel only a short range of 50–100 µm (<∼10 cell diameters). In contrast, β-particles can travel as much as 12 mm, a distance that works well for medium to large tumors but can cause unwanted damage to normal tissues. Auger electrons travel 2–500 nm, making them suitable primarily if the targeting agent can transport the radionuclide into the cell, close to the DNA (5). For these reasons, α-particle therapy shows the most promise for treating micrometastases. However, the nonuniform distribution of RPTs in tumor cell clusters remains a challenge. Thus, methods to use multiple RPT agents that achieve complementary activity distributions within cell clusters are needed to sterilize micrometastases.

MIRDcell version 4 is a multicellular treatment planning software that allows users to simulate responses of cells to different RPTs and formulate the best combinations of these drugs for treatment (6*,*^7^). As previously shown, MIRDcell has been successfully used to predict responses of tumor spheroids to ^225^Ac-DOTA–encapsulating liposomes (^225^Ac-liposomes) that produce differing activity distributions in the spheroids (7). The present work aimed to investigate how MIRDcell can be used to predict the responses of spheroids to cocktails of ^225^Ac-liposomes and [^225^Ac]Ac-DOTA-SCN-trastuzumab (^225^Ac-trastuzumab), an ^225^Ac-labeled monoclonal antibody, and how undertaking such predictions can inform new therapeutic strategies.

The present work with MIRDcell used both published experimental data (1) and new experimental data for human epidermal growth factor receptor 2–positive breast cancer spheroids. The spheroids were exposed to 2 different ^225^Ac carriers: liposomes and trastuzumab, a human epidermal growth factor receptor 2–targeting antibody. These drugs have differing spatial profiles of penetration into the spheroids and different temporal profiles of uptake and clearance in the spheroids. The liposomes were designed to enter tumors and release ^225^Ac-DOTA in response to the slightly acidic pH in the tumor interstitium. The released ^225^Ac-DOTA, which is highly diffusive because of its small size, penetrated deeper into tumors, where antibodies could not reach. On the other hand, the antibodies delivered ^225^Ac primarily to the periphery of the tumors, where ^225^Ac-DOTA that is released from liposomes is cleared quickly. The combination can lead to a more uniform distribution of absorbed dose to the tumor (1).

Predicting the responses of micrometastases via MIRDcell elucidates important factors that affect dose delivery for RPTs. Factors such as migrating ^225^Ac daughter radionuclides (e.g., ^221^Fr, ^217^At, and ^213^Bi), internalization into the cell, irradiation by external sources, and, most importantly, nonuniform penetration through micrometastases may collectively influence the experimental outcomes. In this analysis, we further investigated how these factors may have played a role in the efficacy of an ^225^Ac-based cocktail treatment for micrometastases in vitro and how this treatment may be optimized in future studies.

## MATERIALS AND METHODS

Experimental data for spheroid outgrowths after treatment with ^225^Ac-liposomes, ^225^Ac-trastuzumab, or a combination thereof and their spatiotemporal penetration profiles were previously published by Howe et al. (1). The methods used are briefly summarized as they are relevant to the dosimetry calculations.

### Published Data Used for the Analysis

#### Experimental Spheroid Outgrowths

The tumor-responsive liposomes used by Howe et al. (1) released their payload, ^225^Ac-DOTA, in response to the acidic pH in the tumor interstitium. Human epidermal growth factor receptor 2–targeting antibody, ^225^Ac-trastuzumab, was used at a specific activity of 3.4 ± 0.7 MBq/mg of antibody which corresponds to a molar activity of 5.1 × 10^5 ^GBq/mol. The spheroids comprised the human epidermal growth factor receptor 2–positive human BT474 breast cancer cell line, obtained from the American Type Culture Collection. Spheroids were used for studies once they reached a 400-µm diameter.

For the spheroid outgrowth study, spheroids were incubated, 1 per well of a 96-well plate, in 150 µL of medium for 24 h with ^225^Ac-trastuzumab (10 mg/mL) and for the last 6 h with ^225^Ac-liposomes (1 mM total lipid), reflecting their relative blood circulation times. Five different conditions were evaluated, with varying percentages of activity divided among the liposomes and antibodies (combinations of 0%, 30%, 50%, 70%, and 100% of each carrier) and the total activity concentration kept constant at 13.75 kBq/mL. After incubation, the spheroids were transferred individually to wells with fresh medium and monitored for growth until the nontreated controls stopped growing 17 d later. At that point, the spheroids were individually plated on cell culture–treated, flat-bottomed 96-well plates and allowed to grow. The percentage outgrowth was reported as the number of live cells per well relative to the number of live cells per well for the spheroids that received no treatment, measured after the latter reached confluency (1). We considered outgrowth percentages to be comparable to surviving fractions (SFs).

#### Published Drug Penetration into the Spheroids

Howe et al. (1) incubated the spheroids in medium with Alexa Fluor 647 (Molecular Probes, Inc.)-*N*-hydroxysuccinimide (NHS)-trastuzumab (10 µg/mL) or liposomes (1 mM total lipid) loaded with the fluorescent payload carboxyfluorescein diacetate succinimidyl ester (CFDA-SE liposomes), as surrogates of ^225^Ac-trastuzumab and [^225^Ac]Ac-DOTA, respectively. The spheroids were incubated in medium containing the agents for 24 and 6 h, respectively, reflecting experimental treatment incubation times. At different time points, spheroids were removed from the incubation mixture, frozen, and sliced, and on the equatorial sections, the fluorescence intensities were measured using fluorescence microscopy. The radial concentrations were calculated using an in-house eroding code determining average fluorescence intensity of each 2.5-µm concentric ring in the spheroids’ sections, as previously described (1). This process provided spatiotemporal concentration profiles for both antibodies and liposomes.

### New Experimental Data for Liposome Penetration

Under the rationale that the 18 h of α-particle irradiation by [^225^Ac]Ac-trastuzumab may affect liposome (added at hour 18) penetration, new experiments to study this possibility were performed by the Sofou Laboratory at Johns Hopkins University. Using the methods they published previously (1), we remeasured the CFDA-SE liposome spatiotemporal penetration profiles. However, this time the liposome penetration was measured in spheroids that were first treated with 6.5 kBq/mL ^225^Ac-trastuzumab instead of Alexa Fluor 647-NHS-trastuzumab. As in their published experiments (1), these spheroids were then treated concurrently with CFDA-SE liposomes for the last 6 h.

### MIRDcell Simulation of Experimental Spheroid Data

The MultiDrug feature of MIRDcell was used to simulate responses of 3-dimensional (3D) multicellular clusters that represent spheroids. Spheric 3D clusters consisting of cells with a radius of 6 µm and nuclear radius of 3 µm were used, as was consistent with the BT474 cells (1). The distance between the centers of each cell was set to 12 µm. MIRDcell automatically uses a simple cubic lattice for cells in a cluster (8). By choosing these dimensions, we ensured that the number of cells in the simulated cell clusters was roughly the same as what was reported in the experimental spheroids of the same size (1). Default radiobiologic parameters were used in MIRDcell except for the linear quadratic model’s α-parameter for α-particles, which was set to 2.7 Gy^−1^ on the basis of previous work with this cell line (9).

To account for the absorbed dose to cells from the drugs in the medium during the 24-h treatment period, the full cluster radius was made to be 350 µm. The SF was calculated only for the cells within a 200-µm radius, which was the size of the experimental spheroids. For the ^225^Ac-liposomes, activity was placed on the cell surface as per our prior studies (7). For ^225^Ac-trastuzumab, 100% of the activity was placed either in the cytoplasm or on the cell surface. The spatiotemporal penetration profiles of each drug were uploaded to MIRDcell in the form of time-integrated mean decays per cell at different radial depths obtained through MIRDcell-Ã, a companion software tool that is distributed with the MIRDcell V4 software package (6). Details of the preparation of raw penetration data and time integration are provided in the supplemental materials (available at http://jnm.snmjournals.org). ^225^Ac-liposomes in the spheroids, ^225^Ac-liposomes in the medium, ^225^Ac-trastuzumab in the spheroids, and ^225^Ac-trastuzumab in the medium were modeled as 4 drugs to account for absorbed dose due to drugs in the medium separately. MIRDcell predictions were modeled with and without ^225^Ac daughters.

Since penetration of the liposomes may have increased because of the initial treatment of the spheroids with ^225^Ac-trastuzumab, a comparison using MIRDcell predictions was also made using the CFDA-SE liposome penetration data with and without the pretreatment ^225^Ac-trastuzumab. For predictions with the previously published antibody and liposome penetration data (i.e., without the pretreatment ^225^Ac-trastuzumab), the decays-per-cell radial data from MIRDcell-Ã (based on the total activity concentration of 13.75 kBq/mL for the 100% case) were scaled accordingly for every case using the Amount Multiplier parameter in MIRDcell. For example, in the 50% ^225^Ac-trastuzumab and 50% ^225^Ac-liposome case, the radial data for decays per cell were multiplied by 0.5 for each drug. Predictions were similarly made with the new CFDA-SE liposome penetration data from spheroids with 6.5 kBq/mL ^225^Ac-trastuzumab pretreatment, representing the 50% ^225^Ac-liposome and 50% ^225^Ac-trastuzumab case. However, CFDA-SE liposome penetration data were not collected to represent the 70% ^225^Ac-liposome (30% ^225^Ac-trastuzumab) or the 30% ^225^Ac-liposome (70% ^225^Ac-trastuzumab) case. Thus, the original CFDA-SE liposome penetration profile representing the 100% ^225^Ac-liposome case and the new penetration profile representing the 50% ^225^Ac-liposome case were used to extrapolate the liposome penetration profiles for the 70%–30% and 30%–70% cases. Essentially, weighted averages were used to get to the decays-per-cell radial data for the 70% ^225^Ac-liposome (30% ^225^Ac-trastuzumab) case, and extrapolations were performed to estimate the decays-per-cell radial data for the 30% ^225^Ac-liposome (70% ^225^Ac-trastuzumab) case.

### Optimization of a ^225^Ac-Trastuzumab/^225^Ac-Liposome Cocktail

Within MIRDcell’s MultiDrug tab, there is a 3D Planning feature that acts as artificial intelligence (AI) to determine the optimal molar activities of RPTs used in combination to treat multicellular clusters (6). Accordingly, MIRDcell AI was used to optimize a cocktail of ^225^Ac-trastuzumab and ^225^Ac-liposomes for BT474 spheroids. The penetration profiles of the 50% ^225^Ac-trastuzumab and 50% ^225^Ac-liposomes in spheroids with pretreatment ^225^Ac-trastuzumab (new spatiotemporal data) were used to optimize the cocktail therapy and the ^225^Ac-trastuzumab monotherapy. However, to optimize the ^225^Ac-liposome monotherapy, the penetration profile of the ^225^Ac-liposomes in spheroids without pretreatment ^225^Ac-trastuzumab (old spatiotemporal data) was used. The medium was represented by an extension of the cluster of cells to a 350-µm radius, where a 150-µm shell of cells represents medium. The target SF was set to 0.0001 for the cells in the 200-µm spheroid radius, with the goal of sterilization. The upper limit on the molar activity was set to 10^6^ GBq/mol for both ^225^Ac-trastuzumab and ^225^Ac-liposomes. The lower limit for the antibodies in the cocktail therapy was set to 1.17 × 10^5 ^GBq/mol because that molar activity for the ^225^Ac-trastuzumab in the pretreatment experiment is what produced the ^225^Ac-liposome penetration profile used in the optimization. Optimization was done for ^225^Ac-trastuzumab with and without ^225^Ac daughters and was run to minimize the number of decays either in both medium and spheroid or in just medium. To optimize for ^225^Ac-trastuzumab without ^225^Ac daughters in the spheroids but with ^225^Ac daughters in the medium, the radial decay distributions from MIRDcell-Ã for ^225^Ac-trastuzumab in the spheroids were corrected by a factor of 3.51. This number is reflective of the difference in self-absorbed dose for cells with and without ^225^Ac daughters.

## RESULTS

**FIGURE 1.**
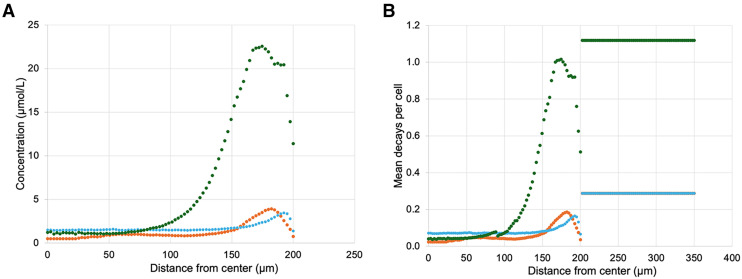
Penetration data in BT474 spheroids (200-µm radius) for Alexa Fluor 647-NHS-trastuzumab (
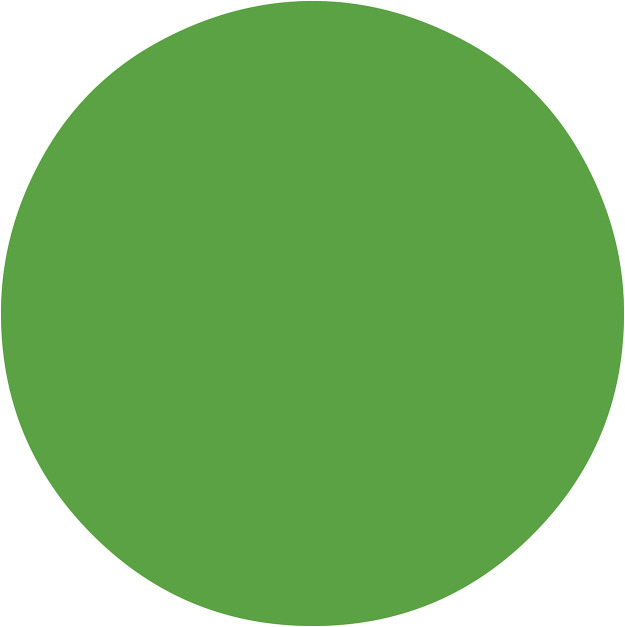
), CFDA-SE liposomes (
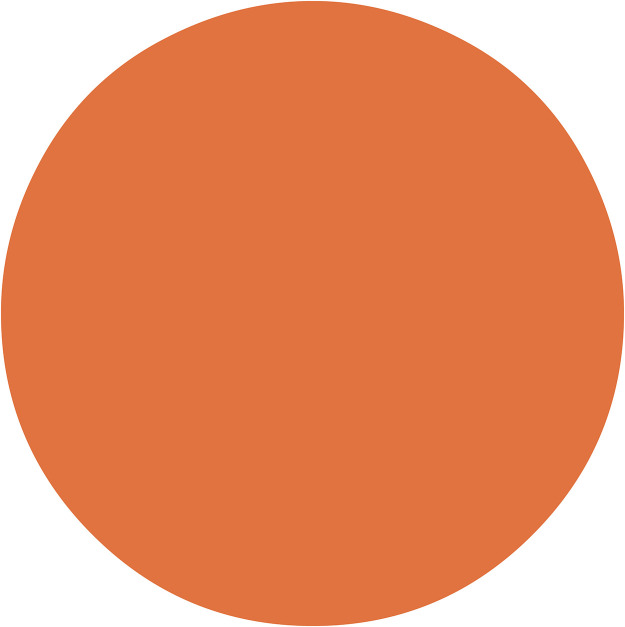
), and CFDA-SE liposomes after preirradiating spheroids with 6.5 kBq/mL ^225^Ac-trastuzumab (
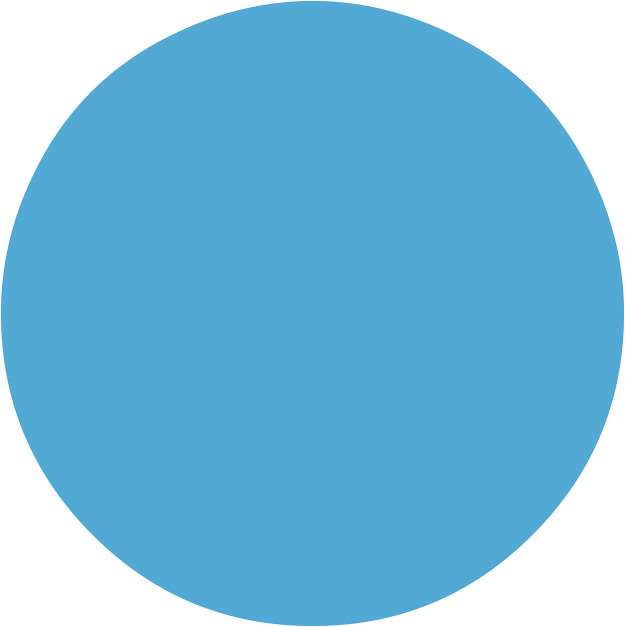
). Shown are time-integrated average concentrations as function of distance from centers of spheroids (A) and correlated mean decays per cell using activity concentrations of 6.875 kBq/mL for all drugs (calculated with MIRDcell-Ã) (B). Distances beyond 200 µm represent medium.

**FIGURE 2.**
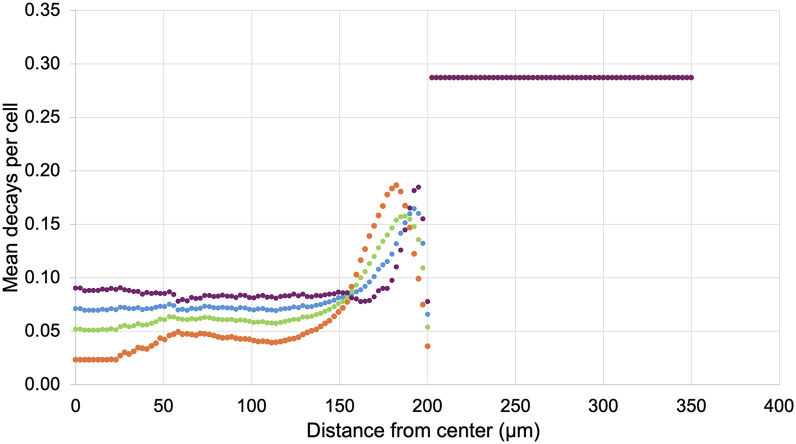
MIRDcell calculated mean decays per cell attributed to liposomes in BT474 spheroids based on experimental CFDA-SE liposome penetration data for 
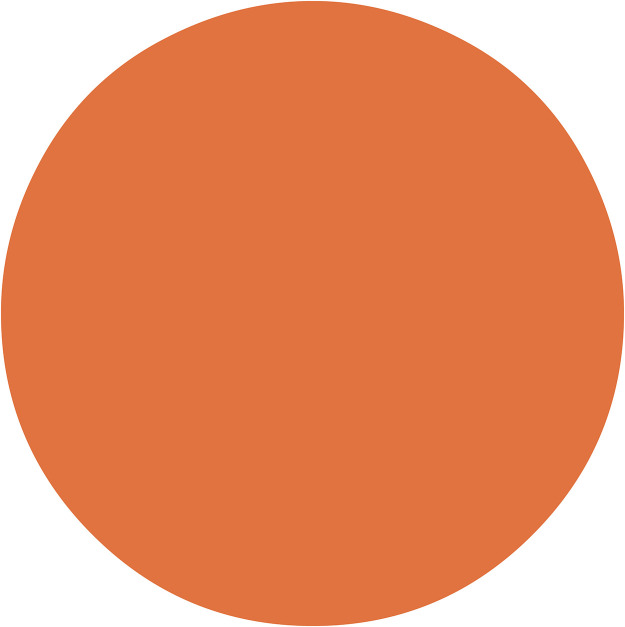
 (100% ^225^Ac-liposome case from Howe et al. (1)) and 
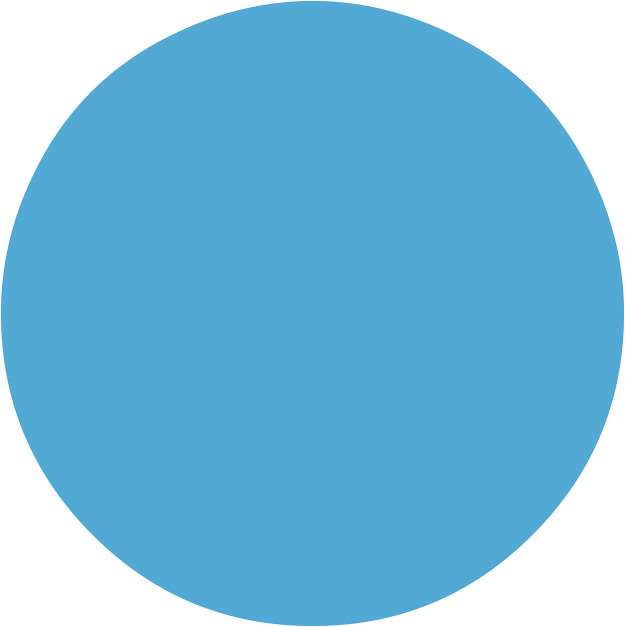
 (50% ^225^Ac-liposome, 50% ^225^Ac-trastuzumab case from newly retrieved data), and extrapolated/averaged (weighted) liposome penetration data for 
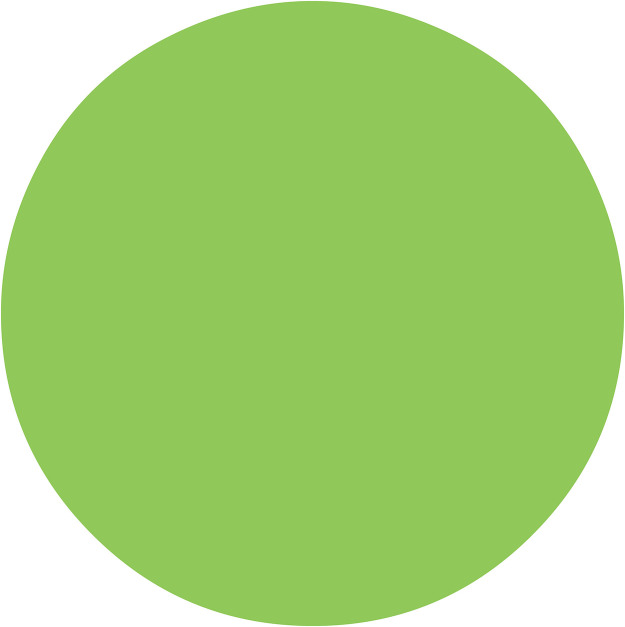
 (70% ^225^Ac-liposome, 30% ^225^Ac-trastuzumab case) and 
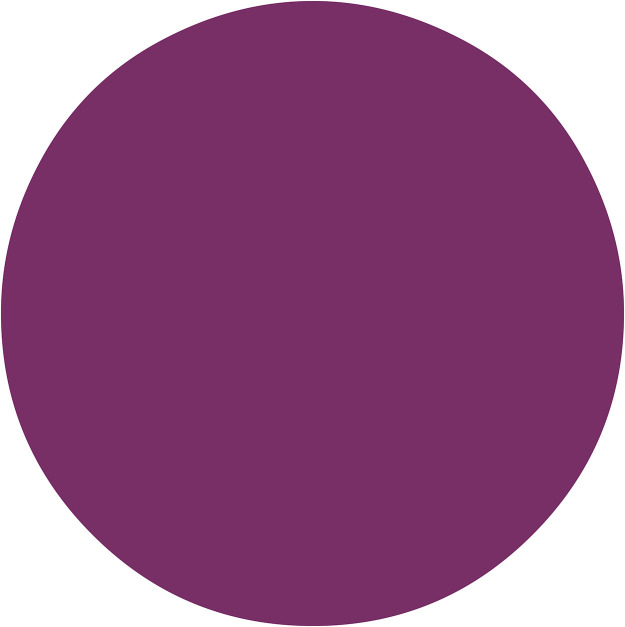
 (30% ^225^Ac-liposome, 70% ^225^Ac-trastuzumab case).

The penetration profiles in Figure 1A show that Alexa Fluor 647-NHS-trastuzumab antibodies accumulated at a much higher concentration in the periphery of spheroids, whereas CFDA-SE liposomes were more equally dispersed throughout the spheroids. Peak accumulation of both Alexa Fluor 647-NHS-trastuzumab antibodies and CFDA-SE liposomes occurred at the periphery of the spheroids within 25 µm from the surface. Notably, the concentration of CFDA-SE liposomes at the center of the spheroids increased when the spheroids were first treated with 6.5 kBq/mL ^225^Ac-trastuzumab (Fig. 1A). This translated to a 42% increase in the total number of decays attributed to ^225^Ac-liposomes between 0 and 100 µm from the centers of the spheroids. The average number of ^225^Ac decays per cell between 0 and 100 µm from the centers of the spheroids for ^225^Ac-trastuzumab, ^225^Ac-liposomes, and ^225^Ac-liposomes with ^225^Ac-trastuzumab treatment were 0.037, 0.050, and 0.071, respectively (Fig. 1B). Figure 2 shows extrapolated penetration profiles of CFDA-SE liposomes for the 70% ^225^Ac-liposome (30% ^225^Ac-trastuzumab) case and the 30% ^225^Ac-liposome (70% ^225^Ac-trastuzumab) case after being converted to mean decays per cell based on the respective activity concentrations for each drug. This extrapolation assumes that the penetration of ^225^Ac-liposomes is proportional to the activity concentration of the ^225^Ac-antibodies during the preirradiation.

The predicted SFs from MIRDcell for the 100% ^225^Ac-trastuzumab case (13.75 kBq/mL) using the average energy spectrum of ^225^Ac with and without daughters in equilibrium with the parent ^225^Ac were 0.039 and 0.209, respectively. The ^225^Ac without-daughters prediction was a better match for the 100% ^225^Ac-trastuzumab case, with average experimental outgrowth being 0.285 (*P* = 0.06, indicating a statistically nonsignificant difference at α < 0.05). Thus, all cases involving ^225^Ac-trastuzumab had predictions made without daughters in the spheroids. The daughters remained in the medium for all predictions. Predicted SFs for the 100% ^225^Ac-liposome case using the average energy spectrum of ^225^Ac with and without daughters were 0.249 and 0.541, respectively. The prediction with daughters was closer to the average experimental outgrowth of 0.275 for the liposomes (*P* = 0.50, indicating a statistically nonsignificant difference at α < 0.05). Thus, we proceeded to include the daughters in the predictions for ^225^Ac-liposomes.

**FIGURE 3.**
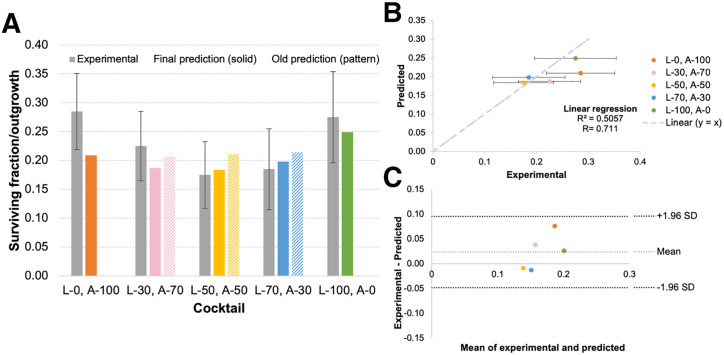
Comparison of BT474 experimental outgrowths taken from Howe et al. (1) to MIRDcell (version 4.14)-predicted SFs as bar plot (A), linear regression plot (B), and Bland–Altman plot (C) (95% limit of agreement, −0.048 to 0.095). Predicted SFs shown were based on ^225^Ac-trastuzumab in spheroids with no ^225^Ac daughters present and ^225^Ac-liposomes in spheroids with ^225^Ac daughters present. ^225^Ac-trastuzumab and ^225^Ac-liposomes in medium both had ^225^Ac daughters present. Bars on abscissa of panel A and legend for panels B and C are labeled to denote percentage of activity corresponding to ^225^Ac-liposomes and ^225^Ac-trastuzumab. For example, “L-100, A-0” is 100% ^225^Ac-liposomes and 0% activity on trastuzumab antibodies. Error bars for experimental outgrowths correspond to SD. Final prediction uses penetration data for CFDA-SE liposomes after spheroids are exposed to 6.5 kBq/mL ^225^Ac-trastuzumab for 50%/50% case; 70% and 30% ^225^Ac-liposome cases use extrapolated/averaged penetration data shown in Figure 2. For CFDA-SE liposomes, published penetration data that were used for old prediction did not involve pretreatment ^225^Ac-trastuzumab. Only final predictions are shown in panels B and C.

**FIGURE 4.**
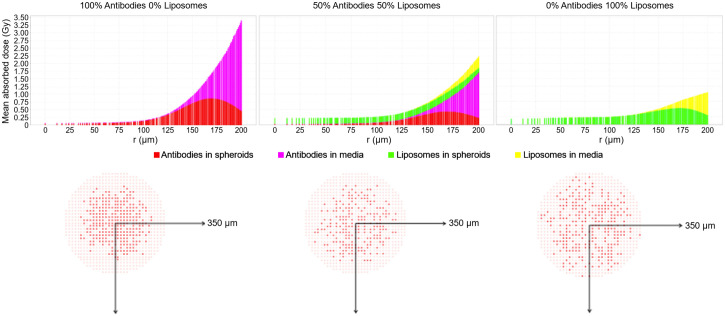
Mean absorbed dose due to ^225^Ac-trastuzumab (antibodies) and ^225^Ac-liposomes (liposomes) in BT474 spheroids and medium for cells at various radial depths within 200-µm-radius spheroids (top). Below each graph, corresponding MIRDcell 3D slice representation of spheroid is shown for equatorial slices of spheroids. Dark red dots in equatorial slices represent living cells, whereas pink dots represent dead cells.

The predicted SFs from MIRDcell shown in Figure 3A for all 5 cases showed no significant differences from the experimental outgrowths when 1-sample *t* tests were applied at an α level of 0.01. Predictions for the 50% ^225^Ac-liposome (50% ^225^Ac-trastuzumab) and 70% ^225^Ac-liposome (30% ^225^Ac-trastuzumab) cases improved when using the CFDA-SE liposome penetration data that were obtained after pretreatment with ^225^Ac-trastuzumab. These were also the predictions that were the closest to the experimental outgrowths (Fig. 3B). The linear regression (Fig. 3B) comparing the MIRDcell predictions with the experiment was not significant (*r*^2^ = 0.5057, *P* = 0.18). However, the Bland–Altman plot (Fig. 3C) showed minimal differences between predicted and experimental results (95% limit of agreement, −0.048 to 0.095) relative to the SD of the experimental outgrowths. These predictions assumed that the ^225^Ac-trastuzumab localized to the cell surface, allowing ^225^Ac daughters to migrate away from the cells. When ^225^Ac-trastuzumab was localized to the cytoplasm, assuming internalization, there was a less than 0.5% difference in all MIRDcell predictions. Further analysis of the absorbed dose attributed to the ^225^Ac-liposomes and ^225^Ac-trastuzumab in the medium revealed that the absorbed dose contribution from the medium is significant for the cells in the periphery of the spheroids (Fig. 4). At a 175- to 200-µm radial distance from the center of the spheroids (i.e., depths of 0–25 µm), about half the absorbed dose comes from the medium for the ^225^Ac-liposomes and more than half for the ^225^Ac-trastuzumab. Figure 4 shows the contrast between the distribution of ^225^Ac-liposomes and ^225^Ac-trastuzumab in the 50%/50% case. Absorbed doses are more equally distributed for ^225^Ac-liposomes than for ^225^Ac-trastuzumab in the spheroids, which peak in absorbed dose at the periphery. This is further highlighted by the MIRDcell 3D slice images in Figure 4, showing more uniform cell death for the 100% ^225^Ac-liposomes than for the 100% ^225^Ac-trastuzumab. They also help visualize cell death concentrated in the periphery of the spheroids for the 100% ^225^Ac-trastuzumab case.

**FIGURE 5.**
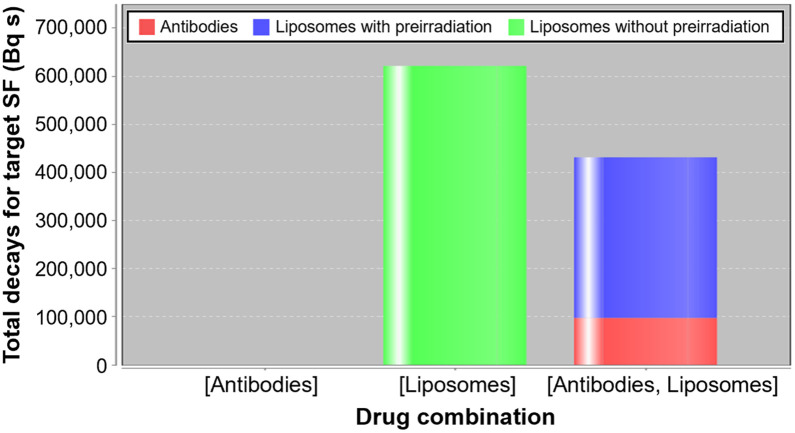
MIRDcell (version 4.16) AI–predicted minimum number of decays necessary to achieve target SF < 0.0001 in BT474 spheroids treated with ^225^Ac-trastuzumab (antibodies), ^225^Ac-liposomes (liposomes), or combination thereof. ^225^Ac daughters were assumed to migrate away from ^225^Ac-trastuzumab and to remain near ^225^Ac-liposomes within spheroid. MIRDcell AI was limited to maximum molar activity of 10^6^ GBq/mol for both drugs and lower limit of 1.17 × 10^5^ GBq/mol for antibodies only. No bar appearing for antibodies indicates that ^225^Ac-trastuzumab alone could not achieve target SF. When optimized molar activities for combined therapy and liposome-only therapy were checked in Monte Carlo–based MIRDcell simulation, zero cell survivors were achieved for both therapies although liposomes required more total decays to achieve same result. “Liposome with preirradiation” indicates use of penetration profiles after preirradiation with ^225^Ac-trastuzumab as in 50%/50% case, and “Liposome without preirradiation” indicates use of liposome penetration profile without preirradiation as in 100% ^225^Ac-liposomes case.

When MIRDcell AI was used to optimize a cocktail of ^225^Ac-liposomes and ^225^Ac-trastuzumab to treat the BT474 spheroids, the final optimization assumed that ^225^Ac daughters migrated away from the ^225^Ac-trastuzumab in the spheroids to be consistent with the MIRDcell predictions. As shown by Figure 5, whereas ^225^Ac-liposomes alone are able to achieve the target SF of 0.0001, they would do so using, minimally, 44% more ^225^Ac decays among the spheroid and medium than would cocktail therapy with ^225^Ac-liposomes and ^225^Ac-trastuzumab (621,319 vs. 431,429 decays). ^225^Ac-trastuzumab alone could not achieve the target SF given the practical limits on molar activity. In the optimized cocktail therapy, ^225^Ac-trastuzumab would be responsible for approximately 23% of the decays, and ^225^Ac-liposomes would be responsible for the other 77%. The optimized molar activities for the cocktail of ^225^Ac-trastuzumab and ^225^Ac-liposomes, respectively, were 1.17 × 10^5^ and 4.40 × 10^4^ GBq/mol. The optimized molar activity for the ^225^Ac-liposomes alone was 8.23 × 10^4 ^GBq/mol. Interestingly, minimizing decays to just the medium produced the same result.

## DISCUSSION

Overall, the results show that the nonuniform penetration profiles of ^225^Ac-trastuzumab and ^225^Ac-liposomes in micrometastases are the most important factor in their efficacy. This finding drives the need to develop a cocktail of these agents rather than to use either agent as a monotherapy. The ^225^Ac-trastuzumab that delivers high absorbed doses to the periphery of the spheroids, coupled with the ^225^Ac-liposomes whose radioactive cargo, ^225^Ac-DOTA, penetrates to the micrometastases’ cores, make for an excellent cocktail therapy that spreads more uniformly through micrometastases.

A significant result was that the CFDA-SE liposomes were able to better penetrate into the spheroids after the pretreatment ^225^Ac-trastuzumab (Fig. 2). The MIRDcell predictions were also better able to match experimental results using the new liposome penetration data (Fig. 3). This finding indicates that optimization of penetration into spheroids, and perhaps metastases, can be achieved by sending a weakly penetrating drug with an α-particle emitter before a second drug that has an enhanced penetrating nature as a result of the first drug. This suggests that consideration must be given to time, duration, and order of drugs used to enhance penetration and RPT efficacy for micrometastases.

Internalization of the radiopharmaceutical into the cells is another factor to consider. Although in many instances one expects radiopharmaceutical internalization into cells to improve their therapeutic efficacy, in the present studies—with cell clusters comprising about 19,000 cells—internalization did not make a big difference in response (SF), as shown by the less than 0.5% difference in all MIRDcell predictions when the ^225^Ac-trastuzumab was assumed to be on the cell surface versus internalized. In these cell clusters, cross-dose from α-particle emitters on nearby cells predominates over self-dose. Hence, the location of the antibody makes a small difference. The difference may be greater if we assume that the daughters did not migrate away from ^225^Ac-trastuzumab. It is conceivable that once the antibody is internalized with ^225^Ac, the daughters may be trapped within the cell. However, internalization’s impact on daughter migration is speculative, as no experimental data were presented by Howe et al. (1) to support including daughters. DOTA is also known to be unable to retain the daughters after bonds are disrupted by the ^225^Ac decay. Furthermore, including the daughters resulted in poor agreement between MIRDcell predictions and experimental spheroid outgrowth. Internalization of α-particle–emitting RPT agents, however, should help treatment efficacy when targeting single circulating or disseminated tumor cells because of the relative increase in cellular S coefficient (i.e., higher absorbed dose per decay yields higher self-dose). Thus, it remains important to find strategies to enhance internalization of α-particle–emitting radiopharmaceuticals, although dehalogenation is a risk in the context of ^211^At (10).

The premise of assuming migrating daughters comes from the DOTA chelator’s inability to trap ^225^Ac daughters after the ^221^Fr recoil after the ^225^Ac α-emission. Liposomes may be better than antibodies at retaining the ^225^Ac daughters (11). This is perhaps why the predictions for the ^225^Ac-liposomes was closer to the experimental result with daughters than without daughters. Perhaps ^225^Ac-trastuzumab’s penetrating only the periphery of the spheroid made it easier for the daughters to escape than did the liposomes’ radioactive cargo, which penetrated more deeply. Yet even without ^225^Ac daughters, the 100% ^225^Ac-trastuzumab prediction overkilled compared with the experimental results. This is most likely because the daughters that were present in the medium from the ^225^Ac-trastuzumab may have plated onto the plastic walls of the cell culture wells that were used during the incubation period. In this case, the contribution to the absorbed dose in the spheroids by radiations emitted by the daughters in the medium would be reduced. These arguments are speculative and point to the importance of collecting detailed quantitative information on daughter activity in all locations at all times throughout experiments. Given the absence of data on daughter distribution, MIRDcell was run in a manner that assumed that daughters were in equilibrium with the parent ^225^Ac whether bound or unbound or not present at all, depending on the particular drug.

Predicting the experimental in vitro results with MIRDcell (Fig. 4) showed that irradiation by radioactivity in the medium can contribute substantially to the absorbed dose at the periphery of the spheroids. This finding was also reported in our earlier work (7). This is an important consideration when translating in vitro success to in vivo applications. Although spheroids are often used as models for micrometastases in ascites fluid, marrow, etc., the pharmacokinetics of drugs in the micrometastases and surrounding tissue can be quite different from those encountered in vitro. For there to be therapeutic success in vivo, the pharmacokinetics in the micrometastases must likely be markedly more favorable than in the normal tissues in which the metastases reside. Caution must be exercised when translating such in vitro findings to in vivo situations.

The present analysis revealed a substantial difference between the absorbed dose from ^225^Ac-trastuzumab and ^225^Ac-liposomes (smaller dose). This difference is due to the significant difference in the number of hours that each was used in treatment and the rapid clearance of ^225^Ac-liposomes compared with ^225^Ac-trastuzumab. When concentrations were translated to mean decays per cell (Fig. 2B), one could appreciate the difference in average mean decays per cell associated with each drug. The more than 3.5 times higher number of decays associated with the ^225^Ac-trastuzumab than with ^225^Ac-liposomes was due to the former’s remaining in the medium for 24 h as opposed to 6 h for the latter. Therefore, the 50%/50% case for equal activities in the medium does not translate to 50%/50% with respect to absorbed doses. Notably, with the MIRDcell AI, we can predict the optimal apportionment in terms of decays from each when used in combination at the corresponding drug concentrations (Fig. 5). The MIRDcell AI translates this to the corresponding molar activities required for each. Although the optimization predictions may be a good estimate, they may be complicated by the changing penetration of the liposomes due to the ^225^Ac-trastuzumab activity in the pretreatment. The optimization results shown for the combinations use the penetration profiles from the 50%/50% case. Ultimately, the penetration profiles may need to be adjusted depending on the magnitude of the pretreatment in terms of time and activity, and this adjustment could be folded into the optimization in the future.

Finally, the most important finding of this MIRDcell AI optimization study is that the best-performing monotherapy, ^225^Ac-liposomes, was able to achieve the target SF of only 0.0001 which is less than 2 cells for spheroids of 200-µm radius, with 44% more total ^225^Ac decays than a cocktail of ^225^Ac-trastuzumab and ^225^Ac-liposomes. Therefore, if we are to prevent reoccurrence of cancers using RPT therapy while minimizing the dose to normal tissue, it is crucial to optimize cocktail therapies to treat micrometastases and single circulating or disseminated tumor cells (12*,*^13^). It is likely that a cocktail of RPTs will be required to fight microscopic disease in cancer.

## CONCLUSION

Predicting responses of micrometastases with MIRDcell may inform experimental approaches using RPT cocktail therapies such as ^225^Ac-DOTA–encapsulating liposomes and ^225^Ac-labeled monoclonal antibodies. It was discovered that penetration of ^225^Ac-DOTA–encapsulating liposomes is enhanced by pretreatment with ^225^Ac-trastuzumab and that strategies to enhance penetration in this way should be explored. As the field of RPT therapy moves forward, strategies to optimize and enhance these factors will become crucial, especially when using a cocktail of RPTs. With thousands of possible combinations of targeting agents and radionuclides, computerized treatment planning capabilities such as those in MIRDcell are needed to help optimize treatment plans with cocktails of RPTs. Cocktails of RPTs are the logical best option for developing cures for metastatic cancers.

## DISCLOSURE

This work was supported in part by NIH 1R01CA245139. MIRDcell is patented under U.S. patent application 2024/0115881 A1, U.S. patent 10,295,543, U.S. patent 9,804,167, U.S. patent 9,623,262, and U.S. patent 8,874,380. No other potential conflict of interest relevant to this article was reported.

## Notes

KEY POINTS **QUESTION:** Can RPT therapy be conducted more effectively and more safely with cocktails of RPTs than with a single RPT? **PERTINENT FINDINGS:** Overall penetration of RPTs into metastases can be improved by first irradiating the periphery of the metastases with an RPT that does not penetrate well and subsequently applying a second RPT. RPT cocktails applied in this manner, as opposed to single agents, may be required to eliminate circulating tumor cells, disseminated tumor cells, and micrometastases. **IMPLICATIONS FOR PATIENT CARE:** Optimized RPT treatment planning for microscopic disease may improve patient outcomes in terms of both effectiveness and safety.
